# Consolidation with carfilzomib, lenalidomide, and dexamethasone (KRd) following ASCT results in high rates of minimal residual disease negativity and improves bone metabolism, in the absence of bisphosphonates, among newly diagnosed patients with multiple myeloma

**DOI:** 10.1038/s41408-020-0297-2

**Published:** 2020-03-02

**Authors:** Maria Gavriatopoulou, Evangelos Terpos, Ioannis Ntanasis-Stathopoulos, Panagiotis Malandrakis, Evangelos Eleutherakis-Papaiakovou, Athanasios Papatheodorou, Nikolaos Kanellias, Magdalini Migkou, Despina Fotiou, Ioanna Dialoupi, Maria Roussou, Nikoletta-Aikaterini Kokkali, Efstathios Kastritis, Meletios A. Dimopoulos

**Affiliations:** 1 Department of Clinical Therapeutics, National and Kapodistrian University of Athens, School of Medicine, Athens, Greece; 2 Department of Medical Research, 251 General Air Force Hospital, Athens, Greece

**Keywords:** Clinical trial design, Haematological cancer

Dear Editor,

Despite the continuous introduction of novel agents in the upfront treatment of multiple myeloma (MM), autologous stem cell transplantation (ASCT) remains a cardinal approach for fit patients^1^. Both the durability and depth of response post ASCT have been recognized as important prognostic factors^2^. Consolidation therapy further improves depth of response. However, there is no consensus on the optimal regimen. Minimal residual disease (MRD) negativity after treatment for newly diagnosed MM patients (NDMM) represents a strong prognostic factor^3^. Therefore, MRD could guide treatment decisions. Βone health is of high importance for NDMM patients as they are at high risk of developing skeletal-related events (SREs) that impair quality of life^4^. Taking all the above factors into consideration, the aim of this prospective study was to evaluate the efficacy, the safety, and the effect on bone metabolism of carfilzomib, lenalidomide, and dexamethasone (KRd) as a consolidation regimen in transplant-eligible patients who had not achieved MRD negativity following ASCT.

This was a prospective assessment of KRd consolidation in NDMM patients who had previously undergone induction, mobilization, stem cell harvest, high-dose melphalan (HDM), and ASCT. The study was conducted in accordance with the Declaration of Helsinki. All patients provided informed consent.

The primary endpoint was to assess efficacy in terms of improving disease response by evaluating depth of response at the beginning and at the end of KRD. The secondary objectives included the percentage of MRD negativity evaluated by Euroflow post KRd, effects on bone metabolism in the absence of bisphosphonates, safety, SRE incidence, progression-free survival (PFS), time to next treatment (TtNT), and overall survival (OS).

All consecutive NDMM patients achieving at least partial response and less than MRD negativity post ASCT were eligible and started consolidation on day 100 post ASCT. Four 28-day cycles of KRd were administered: carfilzomib at a dose of 20 mg/m^2^ iv on cycle 1 day 1 and 56 mg/m^2^ thereafter, on days 1, 8, and 15; lenalidomide at the dose of 25 mg on days 1–21; dexamethasone at 40 mg weekly. Following completion, all patients continued lenalidomide maintenance at 10 mg. Patients did not receive bisphosphonates during or post ASCT, as well as throughout KRd. All patients received prophylaxis with valacyclovir, trimethoprim/sulfamethoxazole, and thromboprophylaxis with low-dose aspirin or low-molecular-weight heparin.

Between January 2018 and June 2019, 40 consecutive patients entered the study. Baseline patient characteristics are depicted in Table 1.

**Table 1:** Baseline characteristics of included patients (*n* = 40).

Variable	Value
Gender (male/female), *n* (%)	21 (53)/19 (47)
Myeloma type, *n* (%)	
IgG	29 (72.5)
IgA	7 (17.5)
Light-chain only	4 (10)
ISS stage, *n* (%)	
I	20 (50)
II	12 (30)
III	8 (20)
R-ISS stage, *n* (%)	
I	13 (32.5)
II	19 (47.5)
III	4 (10)
Induction regimen, *n* (%)	
VRd	20 (50)
VCd	20 (50)
Age (years)	56 (44–67)
PS	
0	38 (95)
1	2 (5)
Cytogenetic abnormalities	Yes/no
Deletion 17p	2/32
T(4;14)	2/33
T(14;16)	0/28
Deletion 13q	9/17
Addition 1q21	7/17
Hb (g/dL)	13.05 (9.6–16.5)
PLTs (×10^9^/L)	236 (97–472)
WBC (×10^9^/L)	5.55 (2.9–8.6)
Neutrophils (×10^9^/L)	3.15 (1–5.3)
Lymphocytes (×10^9^/L)	1.6 (0.6–4.6)
Cr (mg/dL)	0.76 (0.5–1.84)
LDH (U/L)	184 (131–245)
IgG (mg/dL)	857 (453–1930)
IgA (mg/dL)	65.65 (23–314)
IgM (mg/dL)	35 (17.4–97)
Mpeak (g/dL)	0 (0–1.47)
Upeak (mg/24 h)	0 (0–80)
FLC ratio	0.95 (0.03–5.71)
Involved FLC (mg/L)	11.7 (1.47–77.4)

*(R)ISS* (revised) international staging system, *VRd* bortezomib, lenalidomide, dexamethasone, *VCd* bortezomib, cyclophosphamide, dexamethasone, *PS* performance status, *Hb* hemoglobin, *PLTs* platelets, *WBC* whole-blood cell count, *Cr* creatinine, *LDH* lactate dehydrogenase, *FLC* free light chain.

Values for continuous variables are expressed as median (range).

Following ASCT, one (2.5%) patient had achieved stringent complete response (sCR), 4 (10%) were in CR, 30 (75%) were in very good partial response (VGPR), and 5 patients (12.5%) in partial response (PR). All patients in sCR/CR were MRD positive. Post KRd consolidation, 30 out of 38 evaluable patients (79%) pts improved their response status with KRd. Overall, 28 (74%) patients achieved a sCR, one (2.6%) CR, and 9 (24%) VGPR, while 25 (65.8%) patients achieved MRD negativity at the level of 10^–5^. Among the MRD-negative patients, 11 (44%) were R-ISS stage 1, 13 (52%) stage 2, and one (4%) stage 3. [18F]-Fluorodeoxyglucose positron emission tomography–computed tomography (FDG PET/CT) scans were performed in 19 MRD-negative patients; all were negative, except for one.

The markers of bone metabolism were measured in 22 patients with available paired samples at baseline and post KRd (Table 2). TRACP-5b levels showed a significant reduction post consolidation (*p* = 0.011), which reflects a beneficial effect of KRd on bone resorption in the absence of bone-targeting agents. A trend for reduced levels of sclerostin was noted, although not statistically significant.

**Table 2:** Evaluation of markers of bone metabolism at baseline and at the end of KRd consolidation.

Bone marker	Baseline	Post KRd	*p*-value*
*Osteoclast regulators*			
RANKL (pmol/L)	0.13 (0.05, 0.18)	0.14 (0.06, 0.2)	0.721
OPG (pmol/L)	4.45 (3.37, 5.15)	4.32 (3.41, 4.77)	0.673
MIP-1α (pg/ml)	22.39 (15.16, 29.32)	27.09 (16.35, 31.97)	0.322
Activin A (pg/ml)	437.8 (357.7, 507.29)	417.6 (321.6, 474.4)	0.108
*Osteoblast inhibitors*			
Sclerostin (pmol/L)	23.18 (14.4, 27.94)	18.82 (12.82, 21.65)	0.062
DKK-1 (pmol/L)	29.66 (16.09, 38.11)	26.7 (16.24, 38.25)	0.527
*Bone resorption markers*			
CTx (ng/ml)	0.37 (0.23, 0.45)	0.37 (0.15, 0.50)	0.548
Bone TRACP-5b (U/L)	2.45 (2.0, 3.07)	2.02 (1.47, 2.67)	**0.011**
*Bone formation markers*			
bALP (μg/L)	10.02 (5.25, 12.58)	8.42 (4.95, 9.99)	0.158
PINP (pg/ml)	1049 (530, 1318)	994 (480, 1461)	0.858
OC (ng/ml)	11.62 (6.12, 15.21)	13.25 (4.35, 19.24)	0.615

*RANKL* receptor activator of nuclear factor κB ligand, *OPG* osteoprotegerin, *MIP-1α* macrophage inflammatory protein-1α, *DKK-1* Dickkopf-1, *CTX* C-terminal telopeptide, *TRACP-5b* tartrate-resistant acid phosphatase isoform 5b, *bALP* bone alkaline phosphatase, *PINP* procollagen type-I N-propeptide, *OC* osteocalcin.

*Wilcoxon signed-rank test.

Values are expressed as mean (interquartile range).

Bold value denotes statistical significance.

Novel treatment-related toxicities were not reported. Seven patients (17.5%) experienced grade 3 or higher adverse events, including respiratory infections, neutropenia, thrombotic thrombocytopenic purpura, fatigue, pneumonitis, hypocalcemia, γGT, and ALP increase. Unfortunately, one patient died due to septic shock secondary to staphylococcal pneumonia and another due to septic shock secondary to an in-hospital infection on the ground of refractory thrombotic thrombocytopenic purpura complicated by brain hemorrhage. Both patients were on VGPR post ASCT and at the last response assessment. None of them had major comorbidities. No new cases of peripheral neuropathy were noted. Median PFS, TtNT, and OS have not been reached yet.

This prospective study showed that four cycles of KRd consolidation significantly improved depth of response and resulted in a high rate of MRD negativity, along with a positive effect on bone metabolism. Although our study was not designed as a phase 2 trial and the statistical power may be suboptimal, the results are consistent with other studies evaluating the activity of KRd in the newly diagnosed setting^5^. The seminal studies by Jakubowiak et al.^6^ and Korde et al.^7^ provided a strong rationale for evaluating this combination in the frontline setting. However, the number of patients receiving KRd consolidation following ASCT was limited in terms of assessing the isolated effect of consolidation. Furthermore, the Intergroupe Francophone Du MyéLome (IFM) KRd phase II study administered KRd both as the induction regimen and consolidation after ASCT. Among the 41 patients who completed consolidation, 69% achieved sCR/CR and 32/36 (89%) were MRD-negative by NGF^8^. In another phase II trial conducted by the Multiple Myeloma Research Consortium (MMRC), 70 patients completed KRd induction—ASCT-KRd consolidation. Among them, the MRD negativity rate by next-generation sequencing (NGS) combined with CR or better was 67%^9^. More recently, the larger phase 2 FORTE clinical trial evaluated the efficacy of the same treatment schedule. Among 158 patients, NDMM patients who received KRd induction-ASCT-KRd consolidation, the CR or better rate was 60%, and the MRD-negative rate by NGF was 58%^10^. In all these studies, KRd was administered as induction followed by HDM/ASCT and KRd consolidation. In the MMRC trial, lenalidomide and dexamethasone de-escalation was implemented during the consolidation phase^9^. Although cross-trial comparisons present inherent limitations, it seems that KRd results both in higher rates of response improvement and deeper responses compared with other PI and IMiD-based consolidation regimens.

Our study indeed confirms the efficacy of KRd as a consolidation regimen, but with a different and novel proof of concept. Our patient population had received induction treatment with either VRd or VCd instead of KRd, which are considered as the most commonly used upfront regimens; thus, it might be considered as more representative with regard to the real-world clinical practice. Furthermore, we implemented a dosing scheme with once-weekly infusion of carfilzomib in order to assure patient compliance. We have also provided intriguing data on bone metabolism, which are rather scarce in the literature regarding this setting.

Importantly, a major eligibility criterion in our study pertained to the MRD status after ASCT. There are several ongoing trials that use the MRD status as their primary endpoint or formulate the consolidation/maintenance therapeutic strategy based on the MRD status, such as the PERSEUS and the MASTER trials^11,12^. The primary results of the MASTER trial are encouraging, as none of the 27 MRD-negative patients who have entered the observation phase have relapsed during a short-term median follow-up of 5 months. Similar to our approach, the ongoing CONPET study administers KRd consolidation in NDMM patients who have not achieved FDG PET/CT negativity following ASCT^13^. In this context, we propose a risk-adapted strategy based on MRD status for treatment intensification after ASCT.

Regarding bone-specific outcomes, no new SREs were reported. It has to be noted that all patients had achieved VGPR or better post KRd completion. This is in line with the recommendations of the International Myeloma Working Group, suggesting the discontinuation of bisphosphonates for patients who have achieved at least VGPR. Therefore, our results pledge for a bisphosphonate-sparing approach in good responders by administering bone-targeting agents only during the induction phase. Improvement in bone metabolism became evident in our study; KRd consolidation resulted in a significant reduction of the bone resorption marker TRACP-5b, along with a reduction of the osteoblast inhibitor sclerostin. A favorable effect on bone metabolism has also been reported with other PI-based consolidation regimens. Both preclinical and clinical studies have demonstrated the anabolic effects of bortezomib and carfilzomib that counteract the MM-induced deregulation of bone microenvironment^14^. However, the evaluation of bone markers in the consolidation setting should be performed cautiously. The administration of bone- modifying agents during the induction treatment may have a residual beneficial effect, and thus, significant changes in bone markers may not become evident during the consolidation phase^15^. All our patients had received upfront bortezomib-based regimens with bisphosphonates; thus, several bone markers may have reached their plateau levels.

Regarding safety, there were no unanticipated toxicities. Importantly, no serious cardiovascular adverse events and no new cases of peripheral neuropathy were reported. However, there was a high rate of infections observed including two fatal cases. This is in line with a recent meta-analysis of randomized controlled trials, including 1486 patients treated with carfilzomib-based regimens, which showed a 40% excessive risk of serious infections compared with the controls^16^. Similarly, ~10% of the patients experienced at least one severe infectious episode in the primary analysis of the FORTE trial^17^. The inability for vaccination immediately after ASCT and before initiating consolidation may contribute to the increased infection risk. Prophylactic use of granulocyte colony-stimulating factors and/or levofloxacin prophylaxis during the treatment period could be considered.

In conclusion, KRd consolidation with weekly carfilzomib, post ASCT, is highly effective, improves the quality of response by increasing MRD negativity rates, reduces bone resorption, and correlates with the absence of SREs. This triplet combination should be further investigated as a potential consolidation regimen both for standard and high-risk patients.

## References

[1] Ntanasis-Stathopoulos I, Gavriatopoulou M, Kastritis E, Terpos E, Dimopoulos MA (2019). Multiple myeloma: role of autologous transplantation. Cancer Treat Rev..

[2] Lehners N (2018). Analysis of long-term survival in multiple myeloma after first-line autologous stem cell transplantation: impact of clinical risk factors and sustained response. Cancer Med..

[3] Munshi NC (2017). Association of minimal residual disease with superior survival outcomes in patients with multiple myeloma: a meta-analysis. JAMA Oncol..

[4] Terpos E, Ntanasis-Stathopoulos I, Dimopoulos MA (2019). Myeloma bone disease: from biology findings to treatment approaches. Blood.

[5] Landgren O (2019). Carfilzomib with immunomodulatory drugs for the treatment of newly diagnosed multiple myeloma. Leukemia.

[6] Jakubowiak AJ (2012). A phase 1/2 study of carfilzomib in combination with lenalidomide and low-dose dexamethasone as a frontline treatment for multiple myeloma. Blood.

[7] Korde N (2015). Treatment with carfilzomib-lenalidomide-dexamethasone with lenalidomide extension in patients with smoldering or newly diagnosed multiple myeloma. JAMA Oncol..

[8] Roussel M (2016). Frontline therapy with carfilzomib, lenalidomide, and dexamethasone (KRd) induction followed by autologous stem cell transplantation, KRd consolidation and lenalidomide maintenance in newly diagnosed multiple myeloma (NDMM) patients: primary results of the Intergroupe Francophone Du MyéLome (IFM) KRd phase II study. Blood.

[9] Jakubowiak A (2017). High rate of sustained minimal residual disease negativity predicts prolonged survival for the overall patient population in the phase 2 KRd plus autologous stem cell transplantation MMRC trial. Blood.

[10] Gay F (2019). Efficacy of carfilzomib lenalidomide dexamethasone (KRd) with or without transplantation in newly diagnosed myeloma according to risk status: results from the FORTE trial. J. Clin. Oncol..

[11] Sonneveld, P. et al. Bortezomib, lenalidomide, and dexamethasone (VRd) ± daratumumab (DARA) in patients (pts) with transplant-eligible (TE) newly diagnosed multiple myeloma (NDMM): a multicenter, randomized, phase III study (PERSEUS). *J. Clin. Oncol.* **37**(no. 15_Suppl), TPS8055–TPS8055 (2019).

[12] Costa L (2019). Daratumumab, carfilzomib, lenalidomide and dexamethasone (Dara-KRd) induction, autologous transplantation and post-transplant, response-adapted, measurable residual disease (MRD)-based Dara-Krd consolidation in patients with newly diagnosed multiple myeloma (NDMM). Blood.

[13] Terpos E (2013). International Myeloma Working Group recommendations for the treatment of multiple myeloma-related bone disease. J. Clin. Oncol..

[14] Accardi F (2015). Mechanism of action of bortezomib and the new proteasome inhibitors on myeloma cells and the bone microenvironment: impact on myeloma-induced alterations of bone remodeling. Biomed. Res. Int..

[15] Sezer O (2017). Effects of single-agent bortezomib as post-transplant consolidation therapy on multiple myeloma-related bone disease: a randomized phase II study. Br. J. Haematol..

[16] Ball, S., Behera, T., Wongsaengsak, S., Khandelwal, N. & Chakraborty, R. Risk of seriousinfections with carfilzomib in patients with multiple myeloma: a systematicreview and meta-analysis of randomized controlled trials. *Blood* **134** (Supplement_1), 1841 (2019).

[17] Gay F (2018). Carfilzomib-lenalidomide-dexamethasone (KRd) induction-autologous transplant (ASCT)-Krd consolidation vs KRd 12 cycles vs carfilzomib-cyclophosphamide-dexamethasone (KCd) induction-ASCT-KCd consolidation: analysis of the randomized forte trial in newly diagnosed multiple myeloma (NDMM). Blood.

